# Effect of Roasting, Boiling, and Frying Processing on 29 Polyphenolics and Antioxidant Activity in Seeds and Shells of Sweet Chestnut (*Castanea sativa* Mill.)

**DOI:** 10.3390/plants10102192

**Published:** 2021-10-15

**Authors:** Ahmed M. Mustafa, Doaa Abouelenein, Laura Acquaticci, Laura Alessandroni, Rehab H. Abd-Allah, Germana Borsetta, Gianni Sagratini, Filippo Maggi, Sauro Vittori, Giovanni Caprioli

**Affiliations:** 1 School of Pharmacy, University of Camerino, Via Sant’Agostino 1, 62032 Camerino, Italy; ahmed.mustafa@unicam.it (A.M.M.); doaa.abouelenein@unicam.it (D.A.); laura.acquaticci@unicam.it (L.A.); laura.alessandroni@unicam.it (L.A.); germana.borsetta@unicam.it (G.B.); gianni.sagratini@unicam.it (G.S.); sauro.vittori@unicam.it (S.V.); giovanni.caprioli@unicam.it (G.C.); 2 Department of Pharmacognosy, Faculty of Pharmacy, Zagazig University, Zagazig 44519, Egypt; rehabhamed2000@yahoo.com

**Keywords:** *Castanea sativa*, roasting, boiling, frying, HPLC-MS/MS, phenolic compounds, antioxidant activity, seeds, shells

## Abstract

Sweet chestnuts (*Castanea sativa* Mill.) are highly prized nuts, and the consumption of fresh chestnuts is usually preceded by roasting, boiling, and frying. The aim of this work was to simultaneously analyze 29 polyphenolic compounds for the first time in raw, boiled, roasted, and fried chestnut seeds and shells using HPLC-MS/MS. Principal component analysis depending on the HPLC-MS/MS results showed that roasting, boiling, and frying affected the contents of 25 detected phenolic compounds in a unique way, of which the most notable phenolics were gallic acid, ellagic acid, and (+)-catechin. Additionally, total polyphenolic content (TPC) was measured via the Folin–Ciocalteu method, and TPC in seeds and inner and outer shells was increased in all treatments except for microwave-roasted seeds. Furthermore, the higher TPC in the inner and outer shells when compared to seeds supported their higher antioxidant activity (AOA) determined via the DPPH experiment. AOA of seeds was increased in all treatments, while the AOA of shells was higher in roasting and lower in boiling and frying treatments. The assessment of these changes is necessary so that chestnut seed consumption and the recycling of their shells as a natural source of antioxidants can be maximized.

## 1. Introduction

Chestnuts (family Fagaceae) are geographically distributed in three main regions: Europe (*Castanea sativa* Mill.), North America (*C. dentata* Borkh), and Asia (*C. creanata* in Japan, and *C. mollissima* Bl. in Korea and China) [1]. Sweet chestnuts (*Castanea sativa* Mill.) seeds are highly prized nuts in the south of Europe, and they have been common ingredients in the diets of the Mediterranean countries for a long time. Recently, the consumption and use of chestnuts has increased due to the various commercial chestnut products present in the market, for example, frozen nuts, and chestnut purée and flour. However, consumers prefer the fresh products, which are considered to have better quality and greater positive effects on health. The consumption of chestnuts is preceded by boiling, roasting, or frying when they are used in their fresh form [2].

Plants, including medicinal plants and food plants, produce a wide range of bioactive secondary metabolites such as polyphenolic compounds, which are considered an integral part of human diets and are very common bioactive constituents [3]. Polyphenolic compounds have attracted more interest and attention due to their significant health benefits. It has been proven that polyphenols have various biological effects, including prevention of cancer, cardiovascular, and neurodegenerative diseases [4]. Chestnuts have significant amounts of healthy bioactive metabolites, including different classes of antioxidants such as polyphenols (ellagic and gallic acids) [1,5,6,7], ascorbic acid [1,8,9], and carotenoids [1]. The plant dietary antioxidants present in chestnuts may explain their protective role against the oxidative stress common in degenerative diseases [9]. Of the nuts, chestnuts, walnuts, and pecans have been reported to have the highest antioxidant contents [9,10]. One of the most important antioxidant groups in chestnuts is the polyphenols [9]. Brizi et al. [11] reported the neuroprotective role of chestnut polyphenols on human neuroblastoma cells, proving an effect on mitigating neurodegenerative problems. Additionally, chestnuts have become an alternative source of gluten-free flour in cases of celiac disease. Celiac sufferers must use foods which are gluten-free. Chestnut fruits are known to be free from gluten; therefore, many products derived from chestnuts and/or chestnut flour have been created to replace products containing wheat/cereal [1].

The chestnut peeling process generates shells as a waste product. The shells are used as fuel in factories. The valorization of shell residues would improve the economic and environmental aspects of this industrial process. Depending on shell phenolics [12], this type of waste could be exploited as a source of natural antioxidants, because the beneficial health effects associated with their polyphenolics, such as cardioprotective anticarcinogenic, and antimutagenic activities, have been related to their reported antioxidant activity. Therefore, research has focused recently on obtaining antioxidants from natural sources such as plants to replace the harmful synthetic antioxidants that are responsible for many pathological problems such as carcinogenesis and liver damage in animals [13].

Some studies have been performed to evaluate the phenolic composition and antioxidant capacity of chestnut shell waste [12,14,15,16,17]. However, little research has been done on the phenolic composition and antioxidant activity of the edible chestnut seeds [7,8,9,18], and has mostly been limited to fresh or raw nuts. Therefore, scarce data are available regarding the possible changes in phenolic profile and antioxidant capacity of roasted, boiled, and fried chestnuts [5,9], the commonly consumed forms of fresh chestnut. All of the previous studies have dealt with few classes and few components of polyphenolics (primarily ellagic and gallic acids). Therefore, the main aim of the present work was to investigate 29 polyphenolic compounds of different chemical classes, namely phenolic acids, flavan-3-ols, flavonols, dihydochalcone, and flavanone, in raw, boiled, roasted (oven or microwave), and fried chestnut seeds and shells using HPLC-MS/MS. This will help to evaluate and compare the effects of the most commonly used processing methods on the level of each individual polyphenolic compound in order to examine the suitability of processed sweet chestnuts as a natural source of polyphenols. To the best of our knowledge, this work is the first study to use HPLC-MS/MS for simultaneous quantitation of all these bioactive phenolics together in sweet chestnut. In addition, the effect of these processing methods on the total contents of polyphenolics, measured using the Folin–Ciocalteu method, and on the antioxidant activity of chestnut seeds and shells was studied. It is very important to evaluate these changes so that the consumption of sweet chestnuts can be optimized as a natural source of antioxidants. This data will then be utilized as an important part of the ongoing research on optimizing and improving the health benefits of chestnuts.

## 2. Results and Discussion

### 2.1. Effect of Roasting, Boiling, and Frying on Quantities of Individual Phenolics in Chestnut Seeds Determined by HPLC-MS/MS

**Figure 1:**
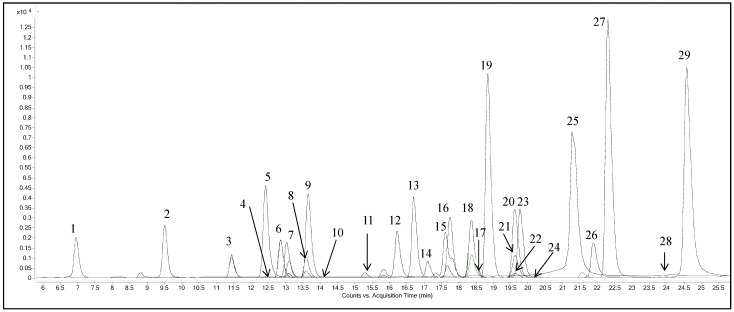
Standard mixture HPLC-MS/MS chromatogram plotted as overlapped transition of each standard in multiple reaction monitoring mode. (1) Gallic acid, (2) neochlorogenic acid, (3) (+)-catechin, (4) procyanidin B2, (5) chlorogenic acid, (6) *p*-hydroxybenzoic acid, (7) (−)-epicatechin, (8) 3-hydroxybenzoic acid, (9) caffeic acid, (10) vanillic acid, (11) syringic acid, (12) procyanidin A2, (13) *p*-coumaric acid, (14) ferulic acid, (15) 3,5-dicaffeoylquinic acid, (16) rutin, (17) hyperoside, (18) isoquercitrin, (19) phloridzin, (20) quercitrin, (21) myricetin, (22) naringin, (23) kaempferol-3-glucoside, (24) hesperidin, (25) ellagic acid, (26) quercetin, (27) phloretin, (28) kaempferol, (29) isorhamnetin.

Before analysis of different chestnut extracts, linearity, limits of quantification (LOQs), limits of detection (LODs), sensitivity, and precision were used to validate the developed HPLC-MS/MS analytical method. The linearity was estimated by constructing calibration curves of the 29 analytes, and all the analyzed compounds showed good linearity. The signal-to-noise ratios of 3:1 and 10:1 were used as criteria for the calculation of the LODs and LOQs, respectively. The LODs ranged from 0.40 to 3.30 µg/L, while the LOQs were in the range of 1.20–10.00 µg/L, showing high sensitivity. The intraday and interday precisions were determined via the injection of the standard mixture solution five times a day, and once a day on three consecutive days, respectively. The developed method showed a great precision as the relative standard deviations (RSDs%) were in the range of 0.34–4.73% in case of interday variations, and ranged from 0.12 to 2.62% for intraday variations. Evaluation of the method specificity was done by setting multiple pairs of precursor/product ions and measuring the stability of retention time, and a high specificity was obtained using HPLC-MS/MS working in dynamic-MRM mode. Retention time stability for each molecule was studied three times over a period of 3 days and expressed by RSDs%, which were in all cases ≤ 1.0%. Similar results were reported in our previous work [19]. The mass spectrometer parameters and the selected ion transitions for the analyzed compounds are reported in Table 1, and the HPLC-MS/MS chromatogram of a standard mixture of the 29 analytes plotted as an overlapped MRM transition of each monitored compound is shown in Figure 1.

Although the total polyphenol contents (TPC) have been evaluated for a broad spectrum of nuts, data regarding the individual phenolic compounds of nuts are still scarce [20]. In the present work, HPLC-MS/MS was used to quantify 29 phenolic compounds in the extracts of fresh, boiled, roasted (oven or microwave), and fried chestnut seeds. Considering the sum of the analyzed compounds, the TPCs were 165.35, 258.18, 103.27, 271.78, and 192.22 mg/kg DW in the raw, boiled, microwave-roasted, oven-roasted, and fried seeds, respectively. TPCs in seeds were increased by all treatments except for microwave roasting; the highest TPC was observed in oven-roasted seeds (271.78 mg/kg) followed by boiled seeds (258.18 mg/kg), and the lowest TPC was detected in microwave-roasted seeds (103.27 mg/kg). The quantitation of all 29 polyphenols by HPLC-MS/MS using polyphenol references was one advantage of this work, because the TPC was measured precisely as the sum of all detected phenolic standards. The TPC or quantification was done using different standards, not by using a single phenolic standard and expressed as standard equivalents as in the traditional spectrophotometric assay. The nonspecificity and overestimation of TPC by spectrophotometric analysis, compared to chromatographic analysis, is due to the interference of nonphenolic substances of the extracts in the spectrophotometric assay. Table 2 shows the phenolic compounds identified in all processed seeds.

#### 2.1.1. Phenolic Acids

Phenolic acids represent about 1/3 of the dietary phenolic compounds detected in plants. Chlorogenic, caffeic, *p*-coumaric, and ferulic acids are examples of hydroxycinnamic acids, while gallic, *4*-hydroxybenzoic, ellagic, syringic, vanillic, and 3,4-dihydroxybenzoic acids are examples of hydroxybenzoic acids [21]. Phenolic acids and their derivatives in plants and foods have received more interest due to their high antioxidant and nutritional properties. Walnut and Brazil nut were reported to have the highest contents of phenolic acid among nuts with values of 0.36 and 0.11 mg/g, respectively, taking into account the scarce data and little characterization work available about this important polyphenol class in most nuts [22]. When including gallic acid (free or bound) with phenolic acids, chestnut, pecan, and almond possess high contents of them in the range of 14 to 900 mg/100 g [22]. Therefore, determination of phenolic acids is important for quality control of chestnuts.

In the current study, 12 phenolic acids were analyzed in the chestnut seed samples, and all of them were detected except for 3,5-dicaffeoylquinic and 3-hydroxybenzoic acid. According to Otles et al. (2012), gallic, ellagic, vanillic, *p*-coumaric acid, chlorogenic, syringic, caffeic, and ferulic acids were determined by HPLC/DAD in 16 different regions in Turkey [23]. In raw chestnut seeds, gallic acid (23.30 mg/kg), followed by ellagic acid (11.11 mg/kg), vanillic acid (9.35 mg/kg), and *p*-coumaric acid (6.73 mg/kg), were the most abundant phenolic acids. Gallic and ellagic acids were previously reported to be the main phenolic acids or polyphenols in sweet chestnuts [1,5,6,7]. The obtained results for gallic and ellagic acids were in a good agreement with the literature [5]; however, their contents are highly variable between different ecotypes, cultivars, and regions [5,7,18,23]. Roasting, boiling, and frying processes affected the phenolic acid compositions of the chestnuts seeds (Table 2). The total phenolic acid content (TPAC) in microwave-roasted, boiled, fried, and oven-roasted chestnut seeds were 44.39, 81.17, 100.61, and 135.88 mg/kg DW, and represented 42.98, 31.44, 52.34, and 50% of total polyphenol contents, respectively, with respect to 59.80 mg/kg DW (36.17%) in the raw sample. In fact, oven-roasted seeds, followed by fried and boiled seeds, possessed higher amounts of ellagic and gallic acids, and TPAC, while microwave-roasted seeds had lower gallic acid content and TPAC in comparison to raw chestnut seeds (Table 2). Gonçalves et al. reported similar results, namely that roasted *C. sativa* possessed higher gallic acid and TPC, and boiled nuts possessed significantly higher contents of gallic and ellagic acids in comparison to raw chestnut seeds [5]. Gallic acid changed from 17.53 to 66.54 mg/kg DW in microwave-roasted seeds and oven-roasted seeds, respectively; ellagic acid varied between 11.11 and 42.80 mg/kg DW in the raw seeds and oven-roasted seeds, respectively; and TPAC ranged from 44.39 to 135.88 mg/kg DW in microwave-roasted seeds and oven-roasted seeds, respectively (Table 2). Increases in gallic acid contents could be explained due to tannin decomposition during oven roasting, frying, and boiling. Additionally, the noticed increments in ellagic acid content after roasting, boiling, and frying are suggested to be due to the release of 3,4,5,30,40,50-hexahydroxydiphenic acid from ellagitannin hydrolysis inside the nuts, which produces ellagic acid [5,24]. Ellagic and gallic acids are the main polyphenolics investigated in sweet chestnuts, and they have several positive health effects, particularly antioxidant, antiplasmodial, and anticarcinogenic activities and the improvement of cardiovascular functions [7,25].

#### 2.1.2. Flavan-3-ols

Flavan-3-ols and their polymers, proanthocyanidin “procyanidins”, form an important phenolic subclass in various nut fruits [20,22]. Procyanidins are considered to be the most abundant polyphenols present in many types of nut, such as hazelnut, almond, pecan, and pistachio. B-type proanthocyanidins are predominant in nuts; however, A-type proanthocyanidins have been shown to occur in various nuts, for example, hazelnut, peanut, and almond [20,22]. Flavan-3-ols such as proanthocyanidins and catechins have been reported to play a crucial role in protection from some health problems, such as cancer and cardiovascular problems, and to possess distinct biological activities, such as antioxidant, angioprotective, and inhibition of platelet aggregation properties [26]

All the current processing procedures decreased the levels of total flavanols in the analyzed seed samples, except for boiling and oven roasting which increased it (1.76 and 1.34 times more, respectively) with respect to the raw sample. The total flavan-3-ol contents in boiled, oven-roasted, fried, and microwave-roasted seeds were 155.05, 117.26, 72.9,9 and 50.58 mg/kg DW, respectively, compared with 87.82 mg/kg DW in raw seeds. (+)-Catechin was the most dominant flavan-3-ol in all of the analyzed samples (Table 2). Boiled chestnut seeds showed the highest amounts of (+)-catechin (139.37 mg/kg), and (−)-epicatechin (7.82 mg/kg), while raw seeds contained the highest amounts of procyanidin B2 (8.67 mg/kg) and procyanidin A2 (0.42 mg/kg). Oven roasting increased the level of (+)-catechin and decreased (−)-epicatechin, which is consistent with Payne et al. [27] who reported that catechin level increased by 640–696% in roasted cacao beans in comparison with the control, and that there was a marked destruction of epicatechin. Increases in the contents of catechin upon roasting were due to the epimerization of (−)-epicatechin to (+)-catechin [27]. The effects of roasting, boiling, and frying methods on the levels of flavanols have never been studied in chestnut seeds, and the obtained results concerning the detection of these compounds in raw chestnut seeds are consistent with previous findings [7,23,26]. The obtained results showed that roasting, boiling, and frying decreased the amounts of procyanidin A2 and procyanidin B2. Roasting generally decreases the proanthocyanidin content of nuts [28]. For example, toasting or roasting pistachios decreases their proanthocyanidin to 12% of that found in the raw nut [29].

#### 2.1.3. Flavonols

Flavonols are considered one of the most important classes among the phenolics (15–20%) in chestnut seeds [30]. Flavonols scavenge reactive oxygen species and inhibit low-density lipoprotein oxidation [30]. In the current study, all nine of the analyzed flavonols (hyperoside, quercitrin, isoquercitrin, rutin, quercetin, kaempferol-3-glucoside, myricetin, isorhamnetin, and kaempferol) were quantified in all seed treatments, and the obtained results were consistent with previous reports for the flavonols detected in raw chestnut seeds and shells [22,30,31]. Hyperoside (2.91 mg/kg) was the major flavonol, whereas isorhamnetin (0.07 mg/kg) was the minor one in the raw seed sample. The total flavonol content (sum of the nine detected flavonols) in the analyzed seed samples was increased by boiling (10.42 mg/kg) and oven roasting (9.63 mg/kg), while microwave roasting (5.52 mg/kg) and frying (8.21 mg/kg) decreased it with respect to the raw sample (8.81 mg/kg). Hyperoside was the predominant flavonol present in all of the analyzed seed samples (Table 2). The highest amount of hyperoside was found in boiled seeds (3.10 mg/kg), followed by fried (3.00 mg/kg) and raw seeds (2.91 mg/kg), whereas the lowest level was found in microwave-roasted seeds (2.09 mg/kg).

#### 2.1.4. Dihydrochalcones

The chestnut seed extracts showed small amounts of dihydrochalcones. The amounts of dihydrochalcones detected in chestnut seeds were affected by the processing method (Table 2). The total dihydrochalcone content (i.e., phloridzin and phloretin) was increased in boiled (11.53 mg/kg), fried (10.42 mg/kg), and oven-roasted (9.01 mg/kg DW) chestnut seeds, and it was decreased in microwave-roasted seeds (2.59 mg/kg DW) with respect to the raw sample (8.92 mg/kg DW). Phloridzin was the major part of these compound contents. The highest amounts of phloridzin were detected in fried seeds (7.78 mg/kg), followed by boiled (7.33 mg/kg), raw (6.82 mg/kg), and oven-roasted (6.54 mg/kg), while the lowest level was found in microwave-roasted seeds (1.69 mg/kg). To our knowledge, this is the first time phloridzin and phloretin have been identified and quantified in chestnut seeds, although they were previously identified in chestnut shells without quantification [31].

#### 2.1.5. Flavanones

The flavanones of interest, i.e., naringin and hesperidin, were not found in chestnut seeds, although they were previously identified in chestnut shells [31], and naringin was reported in chestnut seeds collected only in specific regions in Turkey and not detected in others in the same country [23].

### 2.2. Effect of Roasting, Boiling, and Frying on Quantities of Individual Phenolics in Chestnut Shells Determined by HPLC-MS/MS

In the present work, 29 phenolics were determined by HPLC-MS/MS in the inner and outer extracts of shells of raw, boiled, roasted (oven or microwave), and fried chestnuts, separated from the whole fruit after different processing methods. The phenolic analytes detected in all processed shells are present in Table 2. Taking into account the sum of the detected analytes determined by HPLC-MS/MS, the total phenolic contents (TPCs) were increased in inner and outer shells by oven roasting (773.48, 619.39 mg/kg DW), boiling (764.37, 493.93 mg/kg DW), and frying (634.88, 455.81 mg/kg DW), respectively, while microwave roasting decreased the TPC of inner shells (424.02 mg/kg DW) and showed quite similar amounts in outer shells (296.32 mg/kg DW) compared to the raw inner and outer shells (434.28, 293.66 mg/kg DW, respectively). Generally, the shells showed higher TPC, measured by HPLC-MS/MS, in comparison with their corresponding seeds, and the TPCs in order were: inner shells > outer shells > seeds.

#### 2.2.1. Phenolic Acids

In the current study, all the analyzed 12 phenolic acids were detected in chestnut inner and outer shells except 3,5-dicaffeoylquinic and 3-hydroxybenzoic acid. Gallic acid (118.85, 69.20 mg/kg) followed by ellagic acid (86.35, 35.49 mg/kg), vanillic acid (10, 10.66 mg/kg), and ferulic acid (4.82, 2.37 mg/kg), were the most dominant phenolic acids detected in the inner and outer shells of raw chestnut, respectively. It was observed that the level of gallic acid was higher than that of ellagic acid in both shells, and that the inner shells were richer in gallic and ellagic acids than the outer shells. The current results are consistent with published reports which emphasize that ellagic and gallic acids are the major phenolic acids or polyphenols reported in sweet chestnut shells [12,25,32,33].

The phenolic acid composition of the chestnut shells was affected by roasting, boiling, and frying processes (Table 2). The total phenolic acid contents were increased in the inner and outer shells of boiled (410.33, 233.78 mg/kg), fried (211.24, 225.11 mg/kg), and oven-roasted (277.69, 315.73 mg/kg) chestnut fruits, while they were decreased in the case of microwave roasting (194.76, 97.92 mg/kg) compared to those detected in the inner and outer shells of the raw sample (223.62, 119.17 mg/kg DW). The highest TPAC was detected in the inner shells of boiled fruits (410.33 mg/kg), followed by oven-roasted outer and inner shells (315.73, 277.69 mg/kg), and the lowest content was found in the outer shells of microwave-roasted fruits (97.92 mg/kg). According to the results, the inner shells showed higher total phenolic acid contents than the outer shells in all treatments except in the case of fried and oven-roasted chestnut fruits. Gallic acid content changed from 54.50 to 253.33 mg/kg in microwave-roasted outer shells and boiled inner shells, respectively; meanwhile, ellagic acid varied between 31.42 and 138.10 mg/kg in microwave-roasted outer shells and boiled inner shells, respectively (Table 2).

An additional important finding of the current study is the fact the *C. sativa* wastes have high contents of gallic and ellagic acids when compared to other natural sources [34,35]. Both polyphenols are considered potent antioxidant, anti-inflammatory, anticancer, and antimicrobial agents from natural sources such as plants [32,34]. The commercial production of gallic and ellagic acids is complex and expensive. Therefore, our results showed that *C. sativa* wastes could be an interesting and less expensive alternative [32].

It is noteworthy that the highest levels of gallic, ellagic, and total phenolic acids were detected in inner shells, followed by outer shells and seed. The contents of gallic acid in the inner shells, outer shells, and seeds of raw chestnut fruits were found to be 118.85, 69.20, and 23.3 mg/kg DW; those of ellagic acid were 86.35, 35.49, and 11.11 mg/kg DW; and those of total phenolic acids were 223.62, 119.17, and 59.80 mg/kg DW, respectively. Therefore, the levels of gallic acid, ellagic acid, and total phenolic acids in chestnut outer and inner shell wastes were 3 to 5.1, 3.19 to 7.77, and 2 to 3.74 times more than their corresponding levels in the edible seeds, respectively.

#### 2.2.2. Flavan-3-ols

All the treatment procedures increased the levels of total flavanols in the analyzed inner and outer shells of processed chestnut fruits compared to raw samples. The amounts of total flavanols in inner and outer shells of boiled (248.79, 194.28 mg/kg), fried (369.67, 160.61 mg/kg), oven-roasted (410.24, 170.30 mg/kg), and microwave-roasted (170.99, 138.39 mg/kg) chestnuts were increased compared to those detected in the inner and outer shells of the raw sample (156.85, 119.54 mg/kg DW). Similarly to the seeds, (+)-catechin was the most dominant flavanol in all of the analyzed shell samples (Table 2). The highest amounts of (+)-catechin and (−)-epicatechin were detected in oven-roasted inner shells (350.28 mg/kg) and fried outer shells (11.35 mg/kg); the lowest amounts were found in raw outer shells (91.34 mg/kg) and microwave-roasted inner shells (0.58 mg/kg). These comparative results are in a good agreement with the findings reported by Monagas et al. [20], who mentioned that the levels of (+)-catechin were higher than those of (−)-epicatechin in the skins of different nuts such as peanuts and almonds. In addition, oven-roasted inner shells contained the highest amounts of procyanidin A2 (31.19 mg/kg) and procyanidin B2 (24.56 mg/kg), and boiled outer shells contained the lowest levels i.e., 12.17, 5.47 mg/kg, respectively. In contrast to seeds, procyanidin A2 demonstrated higher concentrations than procyanidin B2 in all shell samples. Previous reports have mentioned that polymers of flavanols are present in skins of almonds and peanuts as both A- and B-type procyanidins, and that the A-type was the most dominant in peanuts whereas the B-type was more predominant in almonds. Additionally, hazelnut was rich in B forms of procyanidins, and the A and B forms of procyanidins are exclusively formed of (epi)catechins [20].

The results showed that the inner (156.85 mg/kg) and outer (119.54 mg/kg) shells of raw fruits were richer in total flavan-3-ols compared to those detected in the corresponding raw seeds (87.82 mg/kg). Similarly, the shells of all processed fruits were more abundant in total flavanols than their corresponding seeds (Table 2). Flavanols are reported to have antioxidant, cardioprotective, anticancer, neuroprotective, and antimicrobial activities [36]. Therefore, chestnut shells could be used as an inexpensive natural source of antioxidants, namely procyanidins and catechins, for application in the food industry and formulation of dietary supplements.

#### 2.2.3. Flavonols

In the present study, all nine of the analyzed flavonols were found in all shell samples, and the obtained results were consistent with previous data for identified flavonols in shells of raw chestnut [31]. The highest total flavonol content was found in the outer shells of oven-roasted (98.60 mg/kg) fruits, whereas the lowest amount was detected in the inner shells of microwave-roasted fruits (22.97 mg/kg). Outer shells showed higher amounts of total flavonol than inner shells; and all the treatments lead to increases in total flavonol contents except in the inner shells of microwave-roasted fruits. Hyperoside, isoquercitrin, myricetin, and rutin were the predominant flavonols in inner shell samples, while hyperoside, isoquercitrin, quercitrin, and isorhamnetin were the most abundant flavonols in outer shells (Table 2). The highest amount of hyperoside was found in oven-roasted outer shells (36.62 mg/kg), while the lowest level was found in fried inner shells (4.22 mg/kg).

In comparison with seeds, the shells showed higher amounts of total flavonols; for example, the outer (46.91 mg/kg) and inner (28.40 mg/kg) shells of raw fruits were richer in total flavonols than the raw seeds (8.81 mg/kg). Similarly, the shells of all processed fruits were more abundant in total flavonols than their corresponding seeds (Table 2).

#### 2.2.4. Dihydrochalcones

The total amounts of dihydrochalcones detected in chestnut shells were positively affected by all cooking procedures, and inner shells showed higher amounts than outer shells (Table 2). The highest content of total dihydrochalcone was detected in boiled inner shells (47.64 mg/kg DW), followed by oven-roasted inner shells (44.26 mg/kg DW), whereas the lowest level was detected in raw outer shells (8.04 mg/kg DW). Phloridzin was the major part of the identified dihydrochalcones. The highest amounts of phloridzin were detected in boiled inner shells, followed by oven-roasted inner shells, while the lowest content was detected in raw outer shells. To our knowledge, this is the first time phloridzin and phloretin have been quantified in chestnut shells, although they were previously identified in chestnut shells without quantification [31].

The chestnut shells showed higher amounts of total dihydrochalcones than seeds; for example, the inner (25.40 mg/kg) and outer (10.33 mg/kg) shells of raw fruits demonstrated higher contents of total dihydrochalcones with respect to raw seeds (8.92 mg/kg). Similarly, the shells of all treated fruits were more abundant in total dihydrochalcones than their corresponding seeds (Table 2).

#### 2.2.5. Flavanones

Similarly to seeds, naringin and hesperidin were not identified in chestnut shells, although they were previously identified in chestnut shells [31].

### 2.3. Effect of Roasting, Boiling, and Frying on Total Polyphenol Content (TPC) Determined by Folin–Ciocalteu Assay

Data from the Folin–Ciocalteu method indicate that nuts possess significant total phenolic contents (TPCs). Sweet chestnut, pistachio, and pecan contain greater than 1000 mg gallic acid equivalents (GAE) /100 g, whereas most nuts have 100 mg GAE /100 g. Therefore, many nuts are among the top dietary sources of polyphenolic compounds [22,37]. The currently obtained results of the TPC in seeds and shells of raw, roasted, boiled, and fried chestnuts are presented in Table 3. The inner shells showed the highest TPC, followed by outer shells and seeds. For example, the TPC of the inner shell (54.04 mg GAE/g DW) of raw chestnut fruit was 1.20 times more than the outer shell (45.01 mg GAE/g DW) and 6.02 times more than the seed (8.98 mg GAE/g DW). It has been reported that polyphenols in nuts are found mainly in the shells, which are discarded after processes such as roasting for the application of seeds in the bakery industry [20]. The current work is a rare study in which the TPCs in seeds, inner shells, and outer shells were compared at the same time, and the obtained results are consistent with previous studies that reported TPC either in seeds [2,5,14,37,38] or in shells [12,25,33]. Roasting, boiling, and frying affected the TPCs of the chestnut seeds and shells (Table 3).

In seeds, the TPC was increased in all treatments except for microwave-roasted seeds which showed the lowest content (8.86 mg GAE/g) compared to raw seeds (8.98 mg GAE/g), while the highest content was detected in oven-roasted seeds (9.43 mg GAE/g), followed by boiled seeds (9.14 mg GAE/g). These results were also consistent with HPLC analyses, which indicated that TPCs in order were: oven-roasted seeds > boiled seeds > fried seeds > raw seeds > microwave-roasted seeds. Similarly, Gonçalves et al. reported that boiling and roasting (in an electric oven) increased TPC compared to raw seeds, and the roasted seeds showed higher TPC than boiled seeds in most of the ten Portuguese cultivars analyzed [5]. Additionally, Wani et al. proved the increase of TPC of chestnut seeds upon roasting [38]. Roasting was reported to decrease the total tannins in *C. sativa*, so the increase in the TPC of roasted *C. sativa* can be explained by the destructive effect of heating on chestnut tannins which leads to generation of smaller phenolic compounds. Moreover, heating causes changes in the chemical structures of certain molecules such as proteins, which are linked to phenolic compounds, and the increases in their ratios may lead to the increases in TPC in plant foods upon heat processing [39]. It was reported that heating resulted in an increase in the TPC of black-eyed peas [40]. However, Neri et al. noticed that chestnuts cured by cold bath showed a higher TPC than raw chestnuts; this may be due to polyphenol diffusion and leakage from shells to the kernels or seeds during the processing [8].

In addition, TPCs in inner and outer shells were positively affected by all current processing methods. In inner shells, TPC varied between 54.04 and 56.82 mg GAE/g in raw inner shells and oven-roasted inner shells, respectively; in outer shells, TPC was in the range of 45.01–46.09 mg GAE/g in raw outer shells and oven-roasted outer shells, respectively (Table 3). The TPC of shells was increased in all treatments except for microwave roasted outer shells (45.03 mg GAE/g) which remained unchanged, in comparison with raw sample (45.01 mg GAE/g).

### 2.4. Effects of Roasting, Boiling, and Frying on the Antioxidant Activity (AOA) of Chestnut Seeds and Shells

Antioxidants occurring in nuts are divided into nutrient and phytochemical (non-nutrient) antioxidants. In addition to well-known nutrient antioxidants such as vitamins A, C, and E, there are several non-nutrient antioxidants such as phenolics and carotenoids in plants. Several non-nutrient antioxidant phytochemicals such as tannins, quercetin, ellagic acid, catechin, chlorogenic acid, and cyanidin, possess strong antioxidant activities. Many previous reports have shown that polyphenolic constituents have higher antioxidant activity than nutrient antioxidants. Most of the antioxidants in all nuts occur in the skin or shells; for example, <10% is present in the walnut after removal of its skin. Furthermore, the AOA decreases considerably when the skin is removed from nuts [22]. In the current study, the effects of roasting, boiling, and frying on the antioxidant capacities of chestnut seeds and shells were determined using the DPPH method, and the obtained values for the analyzed samples are shown in Table 3. In raw chestnut fruits, the inner shell (5.25 mg TE/g DW) demonstrated the highest DPPH value, followed by the outer shell (5.04 mg TE/g DW), while the seed (2.81 mg TE/g DW) exhibited the lowest value. Therefore, the AOA of raw inner and outer shells was 1.87 and 1.79 times higher than that of raw seed.

Roasting, boiling, and frying affected the AOA of the chestnut seeds and shells in different ways. In seeds, the AOA was increased in all treatments in comparison with raw seeds, and the AOA was in the range of 2.8–3.51 mg TE/g DW in raw and boiled seeds, respectively. The increased AOA following all seed treatments (i.e., boiling, frying, oven roasting) except microwave roasting can be attributed to the increases in TPC. Wani et al. reported the enhancement of AOA in microwave-roasted chestnut seed flour [38], and assumed that the short-time, high-temperature conditions of microwave roasting lead to the generation of melanoidin pigments, and these pigments can increase the AOA. Additionally, Woffenden et al. mentioned that products of the Maillard reaction (e.g., melanoidins and reductones) contribute to antioxidant properties upon roasting, particularly in highly heated samples of pale malts [41]. Little millet (*Panicum sumatrense)* also demonstrated enhanced antioxidant activity and TPC upon roasting [42]. In contrast, the AOAs of both inner and outer shells were increased by roasting (microwave and oven) and decreased by boiling and frying. In inner shells, the AOA ranged from 4.75 to 5.39 mg TE/g DW in boiled shells and microwave-roasted shells, respectively; in outer shells, AOA varied between 4.72 and 5.28 mg TE/g DW in fried and microwave-roasted shells, respectively (Table 3). Recently, it was reported that roasting increased the TPC and antioxidant activity of extracts of almond skin compared to oven-drying and blanching; this can be justified by degradation of polymeric polyphenols and/or hydrolysis of glycosidic flavonoids, and aglycone decomposition [20]. The current work proves that chestnut shells possess the greatest antioxidant properties. Therefore, the obtained results show a high potential for recycling chestnut shell byproducts. After suitable processing, these shells can be incorporated into foods and pharmaceutical products with marked health benefits for humans.

### 2.5. Correlation between TPC Determined by Folin–Ciocalteu Assay and Antioxidant Activity

**Figure 2:**
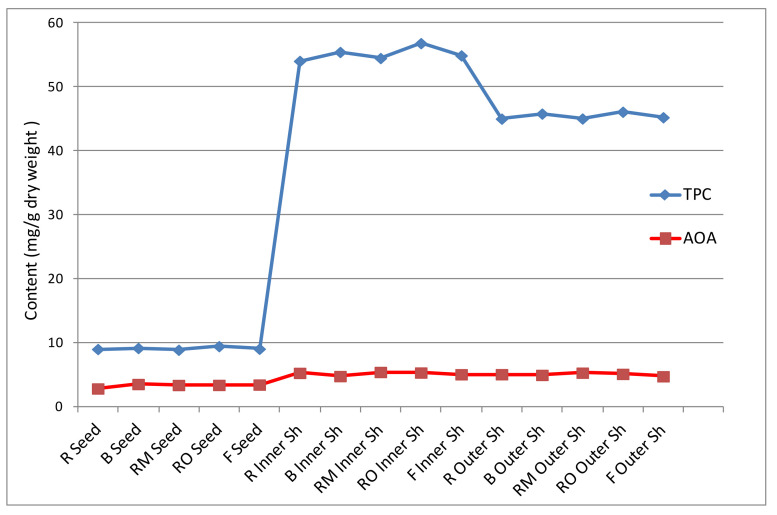
Total phenolic content (TPC) and antioxidant activity (AOA) of seeds and inner and outer shells of chestnut (TPC = mg GAE/g dry weight; AOA = mg Trolox equivalent (TE)/g dry weight). R (raw); B (boiled); RM (roasted, microwave); RO (roasted, oven); F (fried); sh (shell).

**Figure 3:**
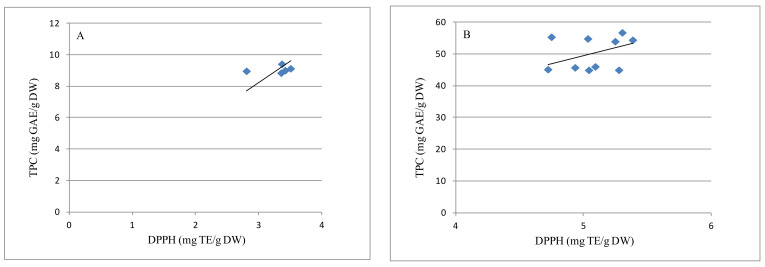
Regression analysis of antioxidant activity (AOA) and total phenolic content (TPC) of seeds (**A**), and shells (**B**) of chestnut (TPC in mg GAE/g dry weight; AOA in mg Trolox equivalent (TE)/g dry weight).

The effects of boiling, roasting, and frying on the AOAs of chestnut seeds and shells were evaluated using the DPPH method. Using the Pearson correlation test, the acquired data were statistically compared to the TPCs calculated in each sample. The relationship between TPCs and AOAs is depicted in Figure 2. AOA showed a high linear correlation with TPC, with good correlation coefficient value (r = 0.97, *p* < 0.01). As shown in Table 3 and Figure 2 and Figure 3, the TPCs of all processed chestnut seeds and shells were well correlated to their radical scavenging activity. Among the examined samples, shells had the highest levels of TPC and the highest AOAs; in particular, the inner shells possessed more AOAs and TPCs than the outer shells. Conversely, seeds demonstrated the lowest amounts of TPC and antiradical property. These findings are consistent with earlier studies that found a link between antioxidant capacity and TPC in chestnut seeds and/or shells; therefore, higher TPCs in chestnut contributed to higher antioxidant activity [8,14,17,25,43]. In addition, different amounts of other antioxidants, such as ascorbic acid, in nuts may contribute to their antioxidant properties, although the major antioxidants in nuts might be polyphenols. For example, Neri et al. reported that the investigated seeds of three chestnut ecotypes showed low polyphenol contents, but were high in ascorbic acid level, which accounted for the discrete antioxidant activity of the nuts [8].

### 2.6. Principal Component Analysis (PCA)

**Figure 4:**
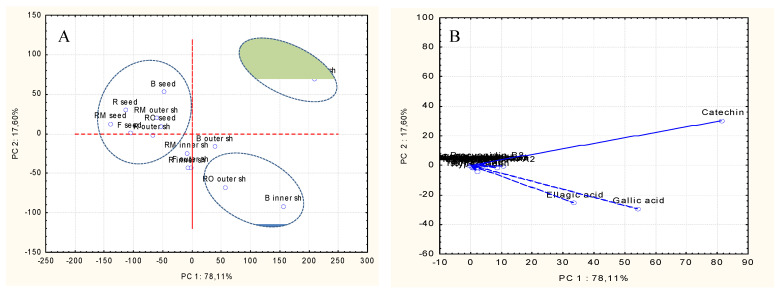
(**A**) The PCA score plot representing the distribution of the roasted, boiled, and fried groups of chestnut seed and shell samples. (**B**) The PCA loading plot demonstrating the correlation between the 25 individual phenolic variables of the chestnut matrix.

PCA enabled us to distinguish between different chestnut treatments based on their individual phenolic components. Figure 4 shows the PCA score and loading plots, as well as correlations between chestnut samples and individual phenolic compounds. A total 95.71% of data variability was explained by the sum of the two principal components (PCs). The fruit processing had a substantial effect on the specific phenolic compounds of chestnut, resulting in three distinct groups, as shown in the graphs. In the first and second PCs, the variance of gallic acid (values of eigenvectors: 54.10, −29.27), ellagic acid (33.43, −25.22), and (+)-catechin (81.44, 30.27) produced the majority of the data variability. The fried inner shell and oven-roasted inner shell samples constitute the group on the right upper side of the score plot. They were mostly characterized by high levels of (+)-catechin. Another group, found on the right lower side of the score plot, was made up of oven-roasted outer shells and boiled inner shells, and correlated with high levels of ellagic and gallic acid. All seed samples and other shell samples were grouped in the left upper part of the score plot, approaching the center, correlated to the other phenolics. Finally, the variability in the data matrix (i.e., individual phenolic compounds) appeared to be linked to the type of processing (i.e., boiling, roasting, and frying) of the samples.

## 3. Materials and Methods

### 3.1. Reagents and Standards

PhytoLab provided analytical standards for kaempferol-3-glucoside and quercetin-3-glucoside (Vestenbergsgreuth, Germany). Sigma-Aldrich provided the reference materials for the remaining 27 of the 29 phenolics (Milan, Italy). Stock solutions of each analyte (1000 mg L^−1^) were made by dissolving pure reference materials in methanol (HPLC-grade) and then keeping them at 5 °C in glass stoppered bottles until analysis. Working solutions of standards at different concentrations were made fresh by diluting stock solutions with methanol (HPLC-grade). Merck provided formic acid at a concentration of 99% (Darmstadt, Germany). Carlo Erba Reagents provided hydrochloric acid (analytical grade) at a concentration of 37% (Milan, Italy). Methanol of HPLC quality was acquired from Sigma-Aldrich, located in Milan, Italy. A Milli-Q SP Reagent Water System filtered deionized water to give ultrapure water with a resistivity of >18 M cm (Millipore, Bedford, MA, USA). Sartorius Stedim provided 0.2 μm polyamide filters which were used to filter all liquids (Goettingen, Germany). Phenex™ RC 4 mm 0.2 μm syringeless filters purchased from the company of Phenomenex, located in Castel Maggiore, BO, Italy, were used to filter all samples before injection into the HPLC instrument.

### 3.2. Plant Material and Sample Preparation

In November 2020, fresh chestnut fruits (*Castanea sativa* Mill.) were purchased from different supermarkets in the city of Camerino, located in the Marche region of Italy. Until extraction, chestnut samples were kept in a freezer at −18 °C. Five groups of chestnuts were created; group 1: raw chestnuts (no treatment); group 2: boiled chestnuts (fruits were boiled in water at a ratio of 1:2, w/v, at 100 °C for 15 min); group 3: roasted in an electric oven (Model: FD 56, Binder, Germany) at 180 °C for 25 min (oven-roasted chestnuts); group 4: roasted using a microwave instrument (Delonghi MWJ63, 900 W, Treviso, Italy) for 4 min (microwave-roasted chestnuts); group 5: fried in a kitchen frying pan for 10 min (fried chestnuts). After cooking processing, the samples (each group weighing 250 g) were peeled to give nuts or seeds and shells, which were separated into inner and outer shells. All samples of the seeds and shells were rapidly cut into little pieces, dried at 45 °C in an electric oven, and then converted into powders using a mortar. Until extraction, the powders were packed in a polyethylene bag and kept at 5 °C. Oven drying at 110 °C until constant weight was reached was used to determine the water content. As a result, the contents of the chemical composition of chestnut seeds and shells were calculated using dry weight (DW).

### 3.3. Extraction Procedures

To extract phenolic compounds, 2 g of powder was sonicated at a frequency of 59 KHz at 25 °C for 60 min with 15 mL of 70% ethanol:water in a FALC ultrasonic bath (Treviglio, Italy). Using a Thermo Scientific IEC CL10 Centrifuge purchased from Thermo Electron Industries SAS (Chateau-Gontier, France), the extraction solutions were centrifuged at 5000 rpm for 10 min before being filtered through a 0.2 m syringeless filter. Finally, the filtered solutions were injected into the triple quadrupole HPLC-MS/MS. Until analysis, all extracts were kept at −18 °C, and the HPLC analysis of each sample was done twice.

### 3.4. HPLC-MS/MS Determination of Individual Phenolic Compounds

The quantification of 29 phenolic analytes was carried out using a modified version of our previously described method [19]. The HPLC-MS/MS investigations were carried out with an Agilent 1290 Infinity series and a Triple Quadrupole 6420 bought from Agilent Technology located in Santa Clara (CA, USA), and linked to an electrospray ionization (ESI) source that operated in negative and positive ionization modes. Using Optimizer Software, the MS/MS parameters of each standard were optimized using flow injection analysis (FIA). The phenolic compounds were separated in gradient elution mode on a Phenomenex Synergi Polar–RP C18 column (250 mm × 4.6 mm, 4 µm) using a mixture of water and methanol as solvents A and B, respectively, both with 0.1%, formic acid. For column protection, a Polar RP security guard cartridge preceded the column (4 mm × 3 mm ID). The mobile phase composition was made up of the following components: 0–1 min, isocratic condition, 20% B; 1–25 min, 20–85% B; 25–26 min, isocratic condition, 85% B; 26–32 min, 85–20% B. A 0.2 μm polyamide filter was used to filter all solutions and solvents. The injection volume was 2 μL and the flow rate was kept at 0.2 mL min^−1^. The temperature of the column was set to 30 °C, and the drying gas temperature in the ionization source was set to 350 °C. The flow rate of the gas was set to 12 L/min, the capillary voltage was 4000 V, and the nebulizer pressure was 55 psi. The peak areas were integrated for quantitation after detection in the dynamic-multiple reaction monitoring (dynamic-MRM) mode. Each analyte’s most abundant product ion was employed for quantification, while the other ions were used for qualitative analysis. Each compound’s unique time window (Δ retention time) was set at 2 min.

### 3.5. Determination of Total Polyphenol Content (TPC) by Folin–Ciocalteu Assay

The TPC was calculated using the Folin–Ciocalteu method given by Mustafa et al. with some adjustments [44]. Briefly, extract solution (0.5 mL) was mixed with 10-fold diluted Folin–Ciocalteu solution (2.5 mL) and 7.5% Na_2_CO_3_ (7 mL) solution. The obtained mixture was held in the dark for 2 h at room temperature and absorption was measured spectrophotometrically at 735 nm using a Cary 8454 UV-Vis (Agilent Technologies, Woburn, MA, USA). A gallic acid calibration curve was used to determine the TPC of the extracts. TPC was measured in mg of gallic acid equivalents (GAE) per gram (g) of dry weight (DW) of plant material. The average of two measurements was used to calculate the results.

### 3.6. Antioxidant Activity (AOA)

The DPPH free radical technique was used to calculate the AOA. The extracts’ free radical scavenging abilities against the DPPH free radical were determined spectrophotometrically using the method reported by Mustafa et al. [19].

To 4.5 mL of 0.1 mM DPPH (ethanolic solution), 0.5 mL of extract solution was added. After mixing, the solution was stored in the dark for 30 min at room temperature before the disappearance of DPPH color was spectrophotometrically measured at 517 nm with an Agilent Cary 8454 UV-Vis spectrophotometer. The results were represented as mg Trolox equivalent (TE)/g dry weight (DW) using Trolox as the reference antioxidant.

### 3.7. Statistical Analyses

#### 3.7.1. Principal Component Analysis (PCA)

To study the relationships between different chestnut samples depending on roasting, boiling, and frying processing, the individual phenolic compound compositions were evaluated using a covariance matrix using PCA which included 25 variables × 15 samples (total data: 375). The software Statistica v. 7.1. from Stat Soft Italia, Vigonza, Italy, was used to construct loading plots and two-dimensional scores for this purpose. The two-dimensional PCA biplot, which included both roasting, boiling, and frying processed samples and individual phenolics, was constructed using eigenvalues calculated using a covariance matrix among 25 individual phenolic compounds as input.

#### 3.7.2. Pearson Correlation Test

The Pearson correlation test was used in Microsoft Excel to determine the correlations between variables (i.e., TPC and AOA) (Microsoft Office 2007). Differences at the *p* < 0.01 level were considered statistically significant. Significant differences were defined as those with *p* values less than 0.01.

The analyses were carried out twice, with the findings given as mean standard deviation (SD).

## 4. Conclusions

Sweet chestnut seeds are highly valued nuts, and consumption of fresh chestnuts is usually preceded by roasting, boiling or frying, which are the most common processing methods. The HPLC-MS/MS method was utilized to simultaneously analyze, for the first time, 29 polyphenolic compounds belonging to the phenolic acids, proanthocyanidins, flavan-3-ols, flavonols, and flavones in raw, boiled, roasted (oven or microwave), and fried chestnut seeds and shells. Principal component analysis based on the HPLC-MS/MS results showed that roasting, boiling, and frying affected the contents of 25 detected phenolic compounds in a unique way, within which the most notable phenolics were gallic acid, ellagic acid, and (+)-catechin. Generally, the shells showed higher contents of total polyphenols, flavan-3-ols, phenolic acids, and flavonols determined by HPLC-MS/MS, in comparison with their corresponding seeds, following the order: inner shells > outer shells > seeds, except for flavonol contents which showed another order: outer shells > inner shells > seeds. Phenolic acids and flavan-3-ols were the major polyphenols in both shells and seeds. Additionally, the TPC of chestnut seeds and shells was measured using the Folin–Ciocalteu method. The inner shells showed the highest TPCs, followed by outer shells and seeds. In seeds, the TPC was increased in all treatment groups except for microwave-roasted seeds, which showed the lowest content compared to raw seeds; the highest content was detected in oven-roasted seeds, followed by boiled seeds. These results were also consistent with HPLC analyses which indicated that TPCs in order were: oven-roasted seeds > boiled seeds > fried seeds > raw seeds > microwave-roasted seeds. In addition, TPCs in inner and outer shells were positively affected by all studied processing methods. Furthermore, antioxidant activity (AOA) evaluated using the DPPH method showed a linear correlation with TPCs of all processed chestnut seeds and shells. Shells contained the highest TPC, and exhibited the greatest AOA; in particular, the inner shells possessed more AOAs and TPCs than the outer shells. Conversely, seeds showed the lowest amounts of polyphenols and antiradical activity. In conclusion, this study confirmed the healthy consumption of roasted, boiled, and fried chestnut seeds and the potential of their shell wastes as a cheap and natural source of polyphenols to be used in nutraceutical and pharmaceutical applications (as supplements or antioxidants).

## Figures and Tables

**Table 1:** Acquisition parameters of HPLC-MS/MS (dynamic-MRM mode) used for the analysis of the 29 phenolic compounds.

No.	Analytes	Precursor ion, *m*/*z*	Product Ion, *m*/*z*	Fragmentor, V	Collision Energy, V	Polarity	Retention Time (Rt, min)
1	Gallic acid	169	125.2 *	97	12	Neg.	6.96
2	Neochlorogenic acid	353	191.2 *, 179	82	12, 12	Neg.	9.52
3	(+)-Catechin	289	245.2 *,109.2	131	8, 20	Neg.	11.44
4	Procyanidin B2	576.99	576.99 *, 321.2	160	0, 32	Neg.	12.41
5	Chlorogenic acid	353	191.2 *, 127.5	82	12, 20	Neg.	12.42
6	*p*-Hydroxybenzoic acid	137	93.2 *	92	16	Neg.	12.86
7	(−)-Epicatechin	289	245.1 *, 109.1	126	8, 20	Neg.	13.03
8	3-Hydroxybenzoic acid	137	93.2 *	88	8	Neg.	13.59
9	Caffeic acid	179	135.2 *, 134.1	92	12, 24	Neg.	13.65
10	Vanillic acid	167	152.4 *, 108.1	88	12, 20	Neg.	14.32
11	Syringic acid	196.9	182.2 *, 121.2	93	8, 12	Neg.	15.28
12	Procyanidin A2	575	575 *, 285	170	0, 20	Neg.	16.18
13	*p*-Coumaric acid	163	119.2 *, 93.2	83	12, 36	Neg.	16.70
14	Ferulic acid	193	134.2 *, 131.6	83	12, 8	Neg.	17.10
15	3,5-Dicaffeoylquinic acid	514.9	353.1 *, 191	117	8, 28	Neg.	17.61
16	Rutin	609	300.2 *, 271.2	170	32, 50	Neg.	17.73
17	Hyperoside	465.01	303 *, 61.1	97	8, 50	Pos.	18.33
18	Isoquercitrin	463	271.2 *, 300.2	155	44, 24	Neg.	18.36
19	Phloridzin	435.39	273 *, 167	155	8, 28	Neg.	18.83
20	Quercitrin	446.99	300.2 *, 301.2	160	24, 16	Neg.	19.61
21	Myricetin	316.99	179.1 *, 182	150	16, 24	Neg.	19.61
22	Naringin	578.99	271.3 *, 151.3	170	32, 44	Neg.	19.62
23	Kaempferol-3-glucoside	447	284.2 *, 255.2	170	24, 40	Neg.	19.77
24	Hesperidin	611.01	303 *, 334.8	112	20, 12	Pos.	20.19
25	Ellagic acid	301	301 *, 229	170	0, 24	Neg.	21.41
26	Quercetin	300.99	151.2 *, 179.2	145	16, 12	Neg.	21.87
27	Phloretin	272.99	167 *, 123	116	8, 20	Neg.	22.30
28	Kaempferol	287.01	153 *, 69.1	60	36, 50	Pos.	23.84
29	Isorhamnetin	314.99	300.2 *, 196.1	145	16, 4	Neg.	24.57

* These product ions were utilized for quantitation; neg., negative; pos., positive.

**Table 2:** Contents of the bioactive phenolics (mg/kg dry weight) determined by HPLC-MS/MS in chestnut seeds and shells.

**No.**	Compound	Seed					Inner Shell					Outer Shell				
		Raw	Boiled	Roasted, Microwave	Roasted, Oven	Fried	Raw	Boiled	Roasted, Microwave	Roasted, Oven	Fried	Raw	Boiled	Roasted, Microwave	Roasted, Oven	Fried
1	Gallic acid	23.30 ± 0.07	49.50 ± 0.13	17.53 ± 0.02	66.54 ± 0.13	43.61 ± 0.01	118.85 ± 0.25	253.33 ± 0.20	106.22 ± 0.11	168.00 ± 0.14	112.24 ± 0.1	69.20 ± 0.03	124.14 ± 0.37	54.50 ± 0.02	153.30 ± 0.23	95.33 ± 0.5
2	Neochlorogenic acid	0.15 ± 0.01	0.18 ± 0.01	0.05 ± 0.03	0.28 ± 0.02	0.13 ± 0.01	0.06 ± 0.03	0.07 ± 0.03	0.02 ± 0.01	0.05 ± 0.01	0.03 ± 0.03	0.04 ± 0.01	0.04 ± 0.01	0.02 ± 0.01	0.04 ± 0.02	0.04 ± 0.01
3	Chlorogenic acid	0.94 ± 0.07	0.63 ± 0.02	0.16 ± 0.02	0.54 ± 0.03	0.66 ± 0.05	0.07 ± 0.05	0.08 ± 0.03	0.02 ± 0.01	0.05 ± 0.01	0.04 ± 0.01	0.06 ± 0.02	0.07 ± 0.01	0.02 ± 0.01	0.07 ± 0.01	0.08 ± 0.02
4	*p*-Hydroxybenzoic acid	1.43 ± 0.10	1.32 ± 0.06	0.16 ± 0.07	1.79 ± 0.05	1.77 ± 0.08	0.51 ± 0.01	0.90 ± 0.03	0.82 ± 0.02	0.32 ± 0.01	0.61 ± 0.01	0.31 ± 0.03	0.53 ± 0.01	0.46 ± 0.01	1.33 ± 0.12	1.88 ± 0.15
5	3-Hydroxybenzoic acid	n.d.	n.d.	n.d.	n.d.	n.d.	n.d.	n.d.	n.d.	n.d.	n.d.	n.d.	n.d.	n.d.	n.d.	n.d.
6	Caffeic acid	3.15 ± 0.01	1.50 ± 0.01	0.19 ± 0.01	3.28 ± 0.01	1.39 ± 0.01	0.73 ± 0.01	0.53 ± 0.01	0.36 ± 0.01	0.14 ± 0.01	0.11 ± 0.01	0.00	0.15 ± 0.01	0.05 ± 0.01	0.16 ± 0.01	0.13 ± 0.01
7	Vanillic acid	9.35 ± 0.04	10.69 ± 0.01	8.02 ± 0.08	13.36 ± 0.08	9.35 ± 0.04	10.00 ± 0.02	9.33 ± 0.04	8.00 ± 0.08	10.66 ± 0.01	8.66 ± 0.06	10.66 ± 0.08	13.33 ± 0.08	9.33 ± 0.04	23.99 ± 0.08	9.60 ± 0.03
8	Syringic acid	0.26 ± 0.01	0.44 ± 0.01	0.35 ± 0.01	0.35 ± 0.01	0.35 ± 0.01	0.96 0.01	0.88 ± 0.01	0.35 ± 0.01	0.70 ± 0.01	0.53 ± 0.01	0.79 ± 0.01	0.88 ± 0.01	0.35 ± 0.01	0.70 ± 0.01	0.61 ± 0.01
9	*p*-Coumaric acid	6.73 ± 0.03	2.28 ± 0.03	0.71 ± 0.01	4.78 ± 0.01	2.96 ± 0.01	1.27 ± 0.08	2.66 ± 0.04	1.83 ± 0.03	1.19 ± 0.03	0.85 ± 0.01	0.25 ± 0.01	0.84 ± 0.01	1.00 ± 0.07	2.38 ± 0.05	2.00 ± 0.02
10	Ferulic acid	3.37 ± 0.01	1.55 ± 0.01	0.25 ± 0.01	2.15 ± 0.01	9.36 ± 0.01	4.82 ± 0.01	4.46 ± 0.03	0.96 ± 0.01	3.86 ± 0.01	3.28 ± 0.01	2.37 ± 0.01	2.04 ± 0.01	0.77 ± 0.01	1.35 ± 0.01	1.13 ± 0.01
11	3,5-Dicaffeoylquinic acid	n.d.	n.d.	n.d.	n.d.	n.d.	n.d.	n.d.	n.d.	n.d.	n.d.	n.d.	n.d.	n.d.	n.d.	n.d. ±
12	Ellagic acid	11.11 ± 0.01	13.09 ± 0.01	16.98 ± 0.03	42.80 ± 0.01	31.02 ± 0.29	86.35 ± 0.56	138.10 ± 0.70	76.17 ± 0.26	92.72 ± 0.07	84.89 ± 0.06	35.49 ± 0.14	91.76 ± 0.52	31.42 ± 0.01	132.41 ± 0.44	121.48 ± 0.13
	Total Phenolic acids	59.80	81.17	44.39	135.88	100.61	223.62	410.33	194.76	277.69	211.24	119.17	233.78	97.92	315.73	225.11
13	(+)-Catechin	74.17 ± 0.13	139.37 ± 0.22	44.28 ± 0.13	108.84 ± 0.20	62.39 ± 0.14	110.93 ± 0.02	207.37 ± 0.31	120.43 ± 0.43	350.28 ± 0.23	316.51 ± 0.81	91.34 ± 0.14	168.35 ± 0.25	109.85 ± 0.55	151.52 ± 0.60	122.17 ± 0.41
14	(−)-Epicatechin	4.56 ± 0.01	7.82 ± 0.06	0.23 ± 0.01	1.66 ± 0.10	4.18 ± 0.07	6.30 ± 0.02	7.27 ± 0.02	0.58 ± 0.01	4.94 ± 0.01	2.99 ± 0.01	7.94 ± 0.04	8.29 ± 0.02	0.75 ± 0.02	8.49 ± 0.09	11.35 ± 0.13
15	Procyanidin A2	0.42 ± 0.02	0.40 ± 0.01	0.17 ± 0.01	0.34 ± 0.06	0.34 ± 0.03	21.63 ± 0.18	19.62 ± 0.11	28.87 ± 0.14	31.19 ± 0.16	27.06 ± 0.12	13.68 ± 0.09	12.17 ± 0.08	20.52 ± 0.17	22.64 ± 0.23	18.61 ± 0.19
16	Procyanidin B2	8.67 ± 0.01	7.46 ± 0.01	5.90 ± 0.04	6.42 ± 0.02	6.07 ± 0.03	17.99 ± 0.02	14.53 ± 0.01	21.10 ± 0.06	24.56 ± 0.02	23.01 ± 0.04	6.57 ± 0.01	5.47 ± 0.01	7.27 ± 0.03	9.65 ± 0.01	8.48 ± 0.03
	Total Flavan-3-ols	87.82	155.05	50.58	117.26	72.99	156.85	248.79	170.99	410.97	369.57	119.54	194.28	138.39	192.30	160.61
17	Rutin	1.59 ± 0.03	1.65 ± 0.03	1.24 ± 0.02	2.05 ± 0.01	1.91 ± 0.01	3.48 ± 0.05	3.56 ± 0.01	2.69 ± 0.07	2.93 ± 0.03	2.87 ± 0.01	2.45 ± 0.06	2.23 ± 0.02	1.61 ± 0.02	1.82 ± 0.01	1.76 ± 0.01
18	Isoquercitrin	2.17 ± 0.01	2.99 ± 0.01	0.83 ± 0.01	2.63 ± 0.05	1.75 ± 0.03	5.24 ± 0.03	11.62 ± 0.02	3.11 ± 0.07	5.01 ± 0.04	4.33 ± 0.03	9.95 ± 0.03	12.68 ± 0.05	10.02 ± 0.02	11.46 ± 0.02	7.21 ± 0.01
19	Hyperoside	2.91 ± 0.01	3.10 ± 0.02	2.09 ± 0.01	3.00 ± 0.03	3.48 ± 0.04	5.45 ± 0.01	10.97 ± 0.08	5.72 ± 0.04	8.81 ± 0.09	4.22 ± 0.06	20.05 ± 0.03	20.55 ± 0.03	23.32 ± 0.21	36.62 ± 0.28	12.62 ± 0.15
20	Quercitrin	0.08 ± 0.01	0.25 ± 0.01	0.05 ± 0.01	0.06 ± 0.001	0.07 ± 0.01	2.72 ± 0.02	3.52 ± 0.02	2.62 ± 0.01	2.62 ± 0.02	1.07 ± 0.01	8.48 ± 0.04	4.12 ± 0.02	7.13 ± 0.07	9.67 ± 0.05	3.34 ± 0.01
21	Myricetin	0.60 ± 0.01	0.85 ± 0.01	0.05 ± 0.01	0.16 ± 0.001	0.36 ± 0.01	4.40 ± 0.05	14.29 ± 0.07	3.00 ± 0.04	8.53 ± 0.03	5.23 ± 0.02	0.57 ± 0.01	1.97 ± 0.02	0.48 ± 0.01	4.75 ± 0.06	2.30 ± 0.04
22	Kaempferol-3-glucoside	0.12 ± 0.01	0.14 ± 0.01	0.03 ± 0.01	0.12 ± 0.01	0.11 ± 0.01	0.18 ± 0.01	0.07 ± 0.01	0.05 ± 0.01	0.05 ± 0.01	0.04 ± 0.01	0.16 ± 0.01	0.09 ± 0.01	0.04 ± 0.01	0.08 ± 0.01	0.07 ± 0.01
23	Quercetin	0.22 ± 0.01	0.25 ± 0.005	0.06 ± 0.01	0.13 ± 0.004	0.10 ± 0.001	0.53 ± 0.01	1.24 ± 0.02	0.37 ± 0.01	1.45 ± 0.01	0.79 ± 0.02	0.79 ± 0.01	3.00 ± 0.02	1.85 ± 0.03	10.46 ± 0.03	5.61 ± 0.02
24	Isorhamnetin	0.07 ± 0.01	0.03 ± 0.01	0.09 ± 0.01	0.27 ± 0.01	0.26 ± 0.01	3.06 ± 0.03	4.67 ± 0.03	1.51 ± 0.02	4.83 ± 0.04	1.58 ± 0.01	4.24 ± 0.01	9.10 ± 0.04	4.15 ± 0.03	23.22 ± 0.16	19.31 ± 0.19
25	Kaempferol	0.98 ± 0.01	1.11 ± 0.03	1.04 ± 0.01	1.18 ± 0.02	0.13 ± 0.01	3.36 ± 0.01	7.69 ± 0.03	3.89 ± 0.06	6.34 ± 0.02	6.41 ± 0.05	0.23 ± 0.01	1.21 ± 0.03	0.30 ± 0.01	0.50 ± 0.01	0.37 ± 0.01
	Total Flavonols	8.81	10.42	5.52	9.63	8.21	28.40	57.62	22.97	40.57	26.55	46.91	54.94	48.90	98.60	52.60
26	Phloridzin	6.82 ± 0.01	7.33 ± 0.02	1.69 ± 0.01	6.54 ± 0.01	7.78 ± 0.01	15.52 ± 0.04	41.83 ± 0.03	28.37 ± 0.11	39.04 ± 0.04	24.68 ± 0.04	4.14 ± 0.01	8.98 ± 0.01	6.69 ± 0.01	7.87 ± 0.01	7.13 ± 0.01
27	Phloretin	2.09 ± 0.03	4.20 ± 0.02	0.90 ± 0.02	2.46 ± 0.05	2.64 ± 0.03	9.88 ± 0.07	5.81 ± 0.01	6.94 ± 0.04	5.22 ± 0.05	2.85 ± 0.02	3.90 ± 0.03	1.94 ± 0.03	4.43 ± 0.02	4.90 ± 0.01	3.19 ± 0.02
	Total Dihydrochalcones	8.92	11.53	2.59	9.01	10.42	25.40	47.64	35.31	44.26	27.52	8.04	10.92	11.12	12.77 ±	10.33
28	Hesperidin	n.d.	n.d.	n.d.	n.d.	n.d.	n.d.	n.d.	n.d.	n.d.	n.d.	n.d.	n.d.	n.d.	n.d.	n.d.
29	Naringin	n.d.	n.d.	n.d.	n.d.	n.d.	n.d.	n.d.	n.d.	n.d.	n.d.	n.d.	n.d.	n.d.	n.d.	n.d.
	Total Flavanones	-	-	-	-	-	-	-	-	-	-	-	-	-	-	-
	Total Polyphenols	165.35	258.18	103.27	271.78	192.22	434.28	764.37	424.02	773.48	634.88	293.66	493.93	296.32	619.39	455.81

n.d., not detectable.

**Table 3:** Contents of total phenolics determined by Folin–Ciocalteu assay, and the antioxidant activity (DPPH) of chestnut seeds and shells.

Parameter	Seed					Inner Shell					Outer Shell				
	Raw	Boiled	Roasted, Microwave	Roasted, Oven	Fried	Raw	Boiled	Roasted, Microwave	Roasted, Oven	Fried	Raw	Boiled	Roasted, Microwave	Roasted, Oven	Fried
Total phenolic content (mg GAE/g DW) ^a^	8.98 ± 0.33	9.14 ± 0.51	8.86 ± 0.36	9.43 ± 0.05	9.02 ± 0.03	54.04 ± 0.27	55.44 ± 0.53	54.49 ± 0.32	56.82 ± 0.76	54.90 ± 0.42	45.01 ±0.17	45.80 ± 0.44	45.03 ± 0.69	46.09 ± 0.57	45.23 ± 0.28
Antioxidant activity (mg TE/g DW) ^b^	2.81 ± 0.58	3.51 ± 0.08	3.36 ± 0.14	3.37 ± 0.38	3.42 ± 0.31	5.25 ± 0.17	4.75 ± 0.84	5.39 ± 0.20	5.30 ± 016	5.04 ± 0.06	5.04 ± 0.45	4.93 ± 0.47	5.28 ± 0.31	5.09 ± 0.73	4.72 ± 0.15

^a^ Contents of total phenolics expressed as mg gallic acid equivalent (GAE)/g dry weight (DW); ^b^ antioxidant activity expressed as mg trolox equivalent (TE)/g dry weight (DW).

## Data Availability

Not applicable.
